# Enhanced Corrosion Resistance of PVD-CrN Coatings by ALD Sealing Layers

**DOI:** 10.1186/s11671-017-2020-1

**Published:** 2017-04-04

**Authors:** Zhixin Wan, Teng Fei Zhang, Ji Cheng Ding, Chang-Min Kim, So-Won Park, Yang Yang, Kwang-Ho Kim, Se-Hun Kwon

**Affiliations:** 1 The Institute of Materials Technology, Pusan National University, Busan, 46241 South Korea; 2 National Core Research Center for Hybrid Materials Solution, Pusan National University, Busan, 46241 South Korea; 3 School of Convergence Science, Pusan National University, Busan, 46241 South Korea; 4 School of Materials Science and Engineering, Pusan National University, Busan, 46241 South Korea; 5 State Key Laboratory of Materials-Oriented Chemical Engineering, College of Chemical Engineering, Nanjing Tech University, Nanjing, 210009 China

**Keywords:** Multilayered hard coating, Hybrid deposition process, Sealing layer, PVD, ALD

## Abstract

Multilayered hard coatings with a CrN matrix and an Al_2_O_3_, TiO_2_, or nanolaminate-Al_2_O_3_/TiO_2_ sealing layer were designed by a hybrid deposition process combined with physical vapor deposition (PVD) and atomic layer deposition (ALD). The strategy was to utilize ALD thin films as pinhole-free barriers to seal the intrinsic defects to protect the CrN matrix. The influences of the different sealing layers added in the coatings on the microstructure, surface roughness, and corrosion behaviors were investigated. The results indicated that the sealing layer added by ALD significantly decreased the average grain size and improved the corrosion resistance of the CrN coatings. The insertion of the nanolaminate-Al_2_O_3_/TiO_2_ sealing layers resulted in a further increase in corrosion resistance, which was attributed to the synergistic effect of Al_2_O_3_ and TiO_2_, both acting as excellent passivation barriers to the diffusion of corrosive substances.

## Background

In industrial applications, damage and failure of numerous metal components result from corrosion. Protecting metals from corrosion is of great technical importance and significance, especially in aggressive environments. One of the most common methods of protection is to deposit protective films or coatings onto metal surfaces [1–3]. A variety of protective ceramic coatings, such as nitrides, carbides, silicides, and transition metal oxides, with relatively high corrosion resistance, wear resistance, and good mechanical strength, have been widely applied in the aviation, aerospace, electronics, petroleum, chemistry, machinery, textile, and automotive industries [4]. Among these protective ceramic coatings, chromium nitride (CrN) has been proven to be one of the most successfully and extensively used coatings in such industries due to its high hardness, excellent wear resistance, and remarkable stability against corrosion [5]. Until now, physical vapor deposition (PVD) techniques have been wildly used for synthesizing such coatings because no toxic chemical precursors are used and no toxic reaction gas or liquid bi-products are produced during the deposition process, which makes PVD to be introduced as an environmental friendly deposition process compared with the thermal chemical vapor deposition (CVD) or even plasma enhanced chemical vapor deposition (PECVD) process [6, 7]. However, due to the line-of-sight transfer of vapor flux during the PVD process, the PVD coatings usually suffer from many intrinsic defects, including columnar structures, pinholes, pores, cracks, and discontinuities, which can significantly affect their corrosion resistance, especially when the substrates are active alloys, such as steel, or exposed to a chloride ion environment [8]. In recent years, to obtain dense microstructure of the coatings and overcome intrinsic defects to improve the corrosion resistance of the coatings, several approaches have been introduced. One such strategy is using more advanced deposition technologies, for example, high power impulse magnetron sputtering (HiPIMS), which exhibits several merits over conventional PVD sputtering, such as increased film density and good adhesion, as well as some advantages over vacuum arc deposition, e.g., free from macroparticles and smooth surface [9]. Another good approach is to add other elements (Si or B) into hard coatings to form nanocomposite coatings with nanosized crystallites surrounded by the matrix [10]. In addition, depositing coatings with multilayered structures can also overcome such intrinsic defects and improve the corrosion properties of hard coatings by synergistic effect of two or more materials [11, 12].

More recently, atomic layer deposition (ALD) techniques have gained great attention for corrosion protection because they are concerned with the requirements of thin film growth, such as uniformity, conformality, low-temperature processing, and exquisite thickness control, and potentially enable high-quality permeation barrier layers [13]. The corrosion protection abilities of ALD thin films, such as Al_2_O_3_, that directly perform on the surface of the substrates or hard coatings to protect the surface or block pinholes and other defects left in the structure have been reported [8, 14]. In our previous work, we demonstrated sandwich-structured coatings of CrN/Al_2_O_3_/CrN obtained using a hybrid deposition process combining HiPIMS and ALD, in which the consecutive ALD-Al_2_O_3_ thin film was inserted into the CrN matrix as a sealing layer. Our previous study showed that the ALD-Al_2_O_3_ film acted as a good insulating barrier with a low defect density and excellent passivation properties to block the diffusion of corrosive substances and improve the corrosion resistance of the CrN hard coatings [12]. However, the Al_2_O_3_ films were originally susceptible to corrosion in water, which means that they were not suitable for use in high humidity conditions or water [15]. In addition, aside from Al_2_O_3_, TiO_2_ is one of the most important reinforcement materials used as a protective layer in engineering materials and offers high strength, good oxidation, and corrosion resistance [4, 16]. Particularly, TiO_2_ is known to display excellent corrosion resistance against aqueous solutions, which makes it a promising candidate to remedy the drawbacks of Al_2_O_3_ sealing layers [17].

In this study, we aimed to further improve the passivation properties and corrosion resistance of the CrN/Al_2_O_3_/CrN hybrid coatings by adding ALD-TiO_2_ layers into the ALD-Al_2_O_3_ sealing layers. A multilayered CrN-Al_2_O_3_/TiO_2_ coating was designed and synthesized with a nanolaminate Al_2_O_3_/TiO_2_ sealing layer (sub-layer thickness of ~2 nm) obtained by alternant deposition of Al_2_O_3_ and TiO_2_ by ALD, which was inserted at the middle position within the thickness of the CrN matrix coating. For comparison, CrN coatings with single sealing layer of Al_2_O_3_ or TiO_2_ were also synthesized. Based on our previous work, the ALD-Al_2_O_3_ layers have shown great passivation properties on highly dense and low-defect hard coatings by HiPIMS; however, the sealing efficiency of the ALD layers on less-dense coatings with pinholes and defects has been rarely studied. Therefore, in this work, relatively porous CrN matrix coatings with rough surfaces were deposited by using a conventional pulsed DC magnetron sputtering technique. The microstructure and corrosion behavior of the multilayered coatings with nanolaminate Al_2_O_3_/TiO_2_ sealing layers in the porous CrN matrix coatings were systematically investigated.

## Methods

The corrosion protection coatings were grown on well-polished stainless steels (SUS304) and Si (100) wafers that were cleaned and degreased by successive rinses in ultrasonic baths of acetone and alcohol for 15 min. The chemical composition of the SUS304 was C (0.044), Si (0.43), Mn (1.12), P (0.032), S (0.004), Ni (8.03), Cr (18.13), N (0.04), and Fe (in wt.%).

### CrN Coating Deposition

The CrN coatings were deposited by using PVD at a temperature of 350 °C. First, a Cr adhesion layer was deposited to improve the coating adhesion. Then, the CrN layers were deposited from a Cr target (99.99%) in Ar (60 sccm) and N_2_ (30 sccm) gas at a working pressure of 4.8 × 10^−3^ Torr by using a pulsed DC sputtering power source, which was held constant at 0.8 kW, and a pulse ratio of 60%. A bias voltage of −100 V was applied to the substrates. The thickness of the CrN layers was controlled by adjusting the deposition time.

### ALD Sealing Layer Deposition

To ensure the least possible contamination at the interface between the ALD and CrN, the as-deposited CrN samples were placed in the ALD chamber as soon as possible after removal from the PVD chamber. The ALD sealing layers, with a thickness of ~20 nm, were deposited on the pre-deposited CrN using a LUCIDA D100 ALD system at a low temperature of 150 °C. The individual Al_2_O_3_ and TiO_2_ sealing layers were obtained by using trimethylaluminum (Al(CH_3_)_3_), titanium isopropoxide (TTIP), and H_2_O reactant, respectively. During the Al_2_O_3_ and TiO_2_ deposition, canisters containing TMA, TTIP, and H_2_O were maintained at temperatures of 25, 60 and 10 °C to achieve a uniform precursor supply. The growth sequence of Al_2_O_3_ consisted of a 0.5 s TMA pulse, 10 s N_2_ purge, 1 s H_2_O pulse, and 10 s N_2_ purge, and of the growth sequence of TiO_2_ included a 0.1 s TTIP assist, 1 s TTIP pulse, 10 s N_2_ purge, 1 s H_2_O pulse, and 10 s N_2_ purge. Here, it was worth to mention that the “assist” step was performed for the TTIP pulse because the vapor pressure of TTIP is relatively low at 60 °C, i.e., a certain amount of N_2_ carrier gas was injected into the canisters for 0.1 s to increase the pressure of TTIP firstly and then released to the chamber for 1 s during the TTIP pulse. This “assist-mode” ensured the sufficient vapor pressure supply of the TTIP precursor during the deposition process. The nanolaminate-Al_2_O_3_/TiO_2_ sealing layers were obtained by repeated sub-cycles of Al_2_O_3_ and TiO_2_, respectively, as mentioned above, whereas the thickness of the unit cycles was fixed at ~2 nm for both, and the number of deposition cycles for each of the unit cycles was calculated using a growth rate per cycle (GPC) of ~1.5 Å/cycle for Al_2_O_3_ and ~0.3 Å/cycle for TiO_2_.

**Fig. 1:**
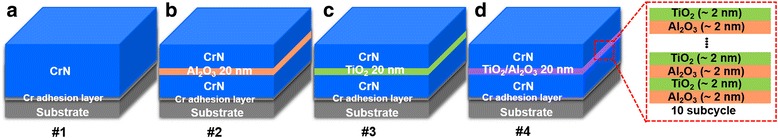
Schematic illustrations of the cross-sectional structure. **a** Pure CrN coating. **b** CrN-Al_2_O_3_; CrN coating with a 20 nm Al_2_O_3_ sealing layer. **c** CrN-TiO_2_; CrN coating with a 20 nm TiO_2_ sealing layer. and **d** CrN-Al_2_O_3_/TiO_2_; CrN coating with a 20 nm nanolaminate-Al_2_O_3_/ TiO_2_ sealing layer

Generally, the pure CrN coatings and the configurations with Al_2_O_3_, TiO_2_, and nanolaminate-Al_2_O_3_/TiO_2_ sealing layers in the CrN coatings were deposited by adjusting the PVD deposition time and ALD deposition cycles, which were named as sample #1 (CrN), sample #2 (CrN-Al_2_O_3_), sample #3 (CrN-TiO_2_), and sample #4 (CrN-Al_2_O_3_/TiO_2_). The schematic illustrations of samples #1 to #4 and the deposition parameters are shown Fig. 1 and listed in Table 1.

**Table 1:** The deposition parameters of the PVD and ALD processes

PVD deposition parameters		ALD deposition parameters			
		Al_2_O_3_		TiO_2_	
Deposition temperature	350 °C	Precursor (TMA)	Cooling; 10 °C	Precursor (TTIP)	Heating; 60 °C
Deposition time	2 h	Reactant (H_2_O)	Cooling; 10 °C	Reactant (H_2_O)	Cooling; 10 °C
Working pressure	4.8 × 10^−3^ Torr	Purge gas	N_2_; 50 sccm	Purge gas	N_2_; 50 sccm
Ar flow rate	60 sccm	Deposition temperature	150 °C	Deposition temperature	150 °C
N_2_ flow rate	30 sccm	Line temperature	100 °C	Line temperature	100 °C
Bias voltage	100 V	Growth rate	1.5 Å/cycle	Growth rate	0.3 Å/cycle

### Coating Characterization

An x-ray diffractometer (XRD, D8-Discovery Brucker, 40 kV) with 1.54 Å Cu-Kα radiation was used to identify the crystal structure of the films. The surface and cross-section micrographs of the coatings without and with ALD sealing layers were evaluated using scanning electron microscopy (SEM, Hitachi, S-4800, 15 KV) and atomic force microscopy (AFM, asylum research MFD-3D). The CrN-Al_2_O_3_/TiO_2_ sample was chosen to further evaluate the ALD sealing effect of the CrN using transmission electron microscope (TEM, TALOS F200X) with energy dispersive spectrometer (EDS) after focused ion beam (FIB) sample preparation. The electrochemical properties of the coatings without and with ALD sealing layers were investigated by a three-electrode electrochemical cell using electrochemical workstation (Princeton, VersaSTAT 4). The potentiodynamic and potentiostatic polarization tests of the samples were obtained in 3.5 wt.% solutions of NaCl at room temperature. A saturated calomel electrode (SCE) and platinum (Pt) mesh were used as the reference electrode and the counter electrode, respectively. All given potentials were reported vs. the SCE. The measurement range was from −1.0 to 1.2 V during the potentiodynamic tests, and the potential was fixed at 0.4 V for the potentiostatic tests because 0.4 V was the pitting region of the SUS304 substrate, which was reported in our previous study [12].

## Results and discussion

**Fig. 2:**
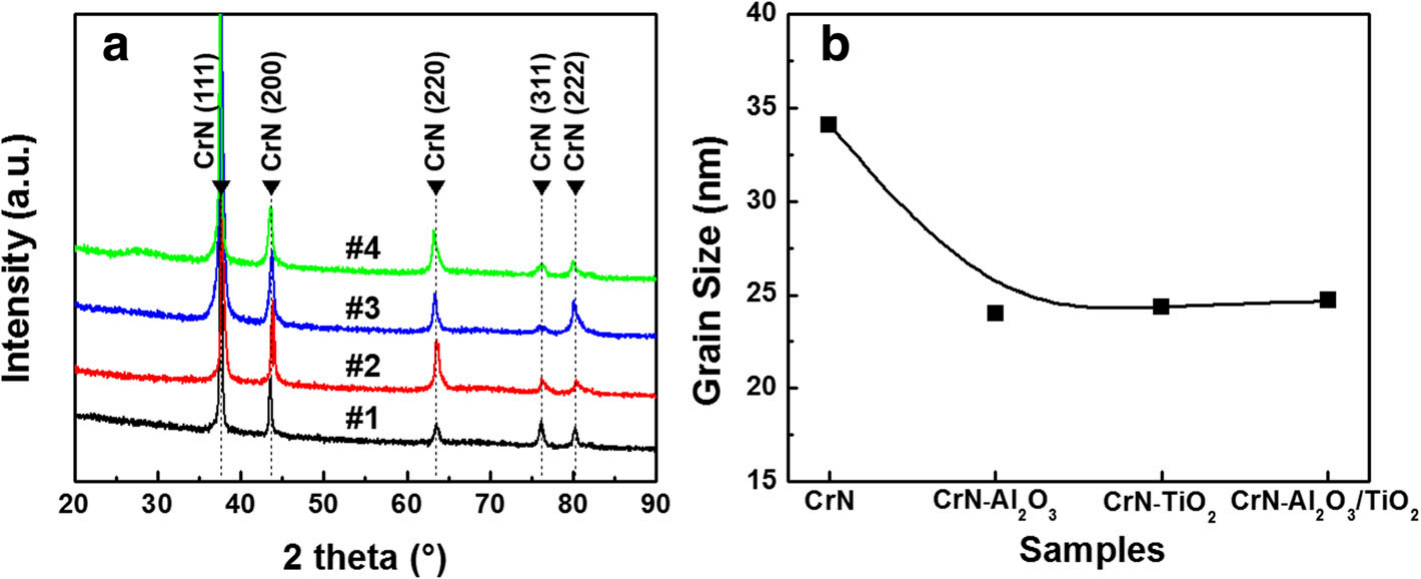
**a** XRD patterns and **b** average grain size of the pure CrN and CrN hybrid coatings with various sealing layers (CrN-Al_2_O_3_, CrN-TiO_2_, and CrN-Al_2_O_3_/TiO_2_)

X-ray diffraction patterns of the as-deposited CrN and the multilayered coatings of CrN-Al_2_O_3_, CrN-TiO_2_, and CrN-Al_2_O_3_/TiO_2_ are shown in Fig. 2a. The diffraction patterns exhibited a cubic phase within the crystalline CrN film with mixed orientations of (111), (200), (220), (311), and (222) crystal planes. No XRD peaks corresponding to other crystalline phases, such as Al_2_O_3_, TiO_2_, or other CrN phases, were observed, indicating the sealing layer had an amorphous structure and the insertion of the sealing layer did not provoke the phase transformation of the CrN matrix. The grain size was calculated by using Williamson-Hall method because it provides a better approach for estimating *D* than Scherrer’s formula, as reported previously [18]. As shown in Fig. 2b, the grain size of the multilayered coatings (approximately 24 nm) significantly decreased after inserting the sealing layer compared with that (approximately 34 nm) of the as-deposited CrN, which was attributed to the sealing layer interrupting the growth of the original CrN and the new nucleation of CrN grains regrowing on the ALD-modified surface during the second deposition process.

**Fig. 3:**
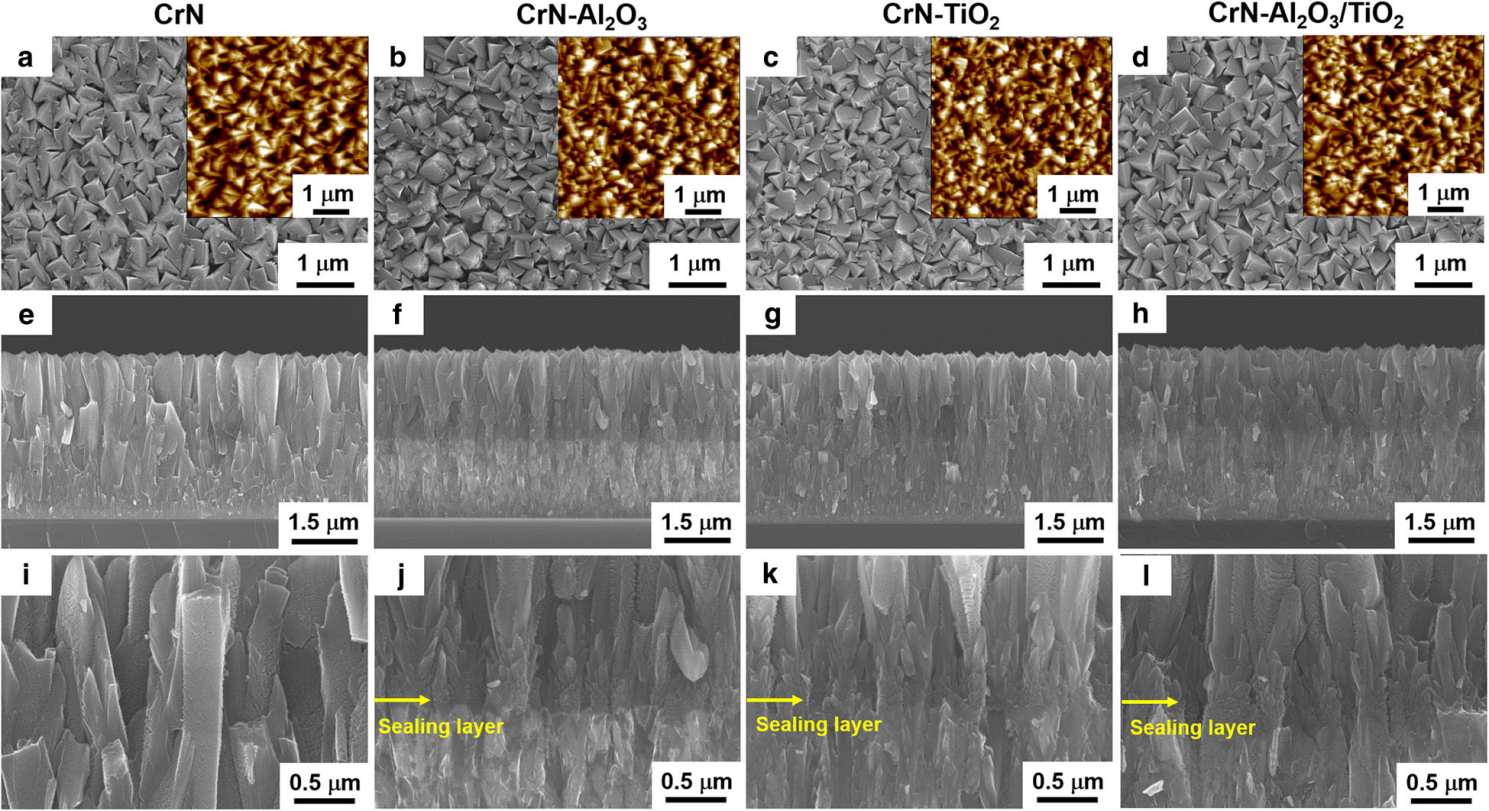
SEM surface images and cross-sectional images of the pure CrN and CrN hybrid coatings with various sealing layers. **a**, **e**, **i** CrN. **b**, **f**, **j** CrN-Al_2_O_3_. **c**, **g**, **k** CrN-TiO_2_. **d**, **h**, **l** CrN-Al_2_O_3_/TiO_2_. The corresponding AFM images were inserted in Fig. 3a–d

Figure 3 presents the top view and cross-sectional images of the as-deposited CrN and multilayered coatings of CrN-Al_2_O_3_, CrN-TiO_2_, and CrN-Al_2_O_3_/TiO_2_ by SEM observation. The as-deposited CrN exhibited a pyramid-like surface with a porous columnar structure, and the pinhole defects were clearly observed, as seen in Fig. 3a, e, and i, which consisted of long columnar grains and clear grain boundaries throughout the whole coating (approximately 3.5 μm). In contrast, the multilayered coatings showed denser and shorter columnar grain structures where the location of the sealing layer could be clearly confirmed. The interrupted morphologies indicated an intergranular fracture along the columnar grains, in which fine granular grains were developed throughout the coatings due to the new nucleation of the CrN on the modified surface after ALD sealing layer deposition. No obvious morphology changes were observed among the hybrid coatings with different sealing layers (Fig. 3f–h). Although pinhole defects could also be seen on the top of the CrN surface of the ALD sealed samples, it was expected that the ALD sealing layer could cover the pore walls along the grain boundary of CrN near the substrate. The cross-sectional observation of the CrN-Al_2_O_3_ shown in Fig. 3f, j exhibited the different contrast between the top CrN and bottom CrN, which was considered due to the successful deposition of the ALD oxide sealing layer, and this phenomenon with relatively weak contrast was also confirmed in the CrN-Al_2_O_3_/TiO_2_ specimen in Fig. 3h, l. The images inserted in Fig. 3a–d show the surface morphologies of the as-deposited CrN and multilayered coatings of CrN-Al_2_O_3_, CrN-TiO_2_, and CrN-Al_2_O_3_/TiO_2_ investigated by AFM. The acquired 5 μm × 5 μm images of the surface topography of these coating are presented. The root-mean-square (RMS) values of all specimens were approximately 60 nm, and no obvious changes of the RMS value were observed after the sealing layer insertion. However, based on the SEM and AFM analysis, a decrease in the grain size of the multilayered coatings (Fig. 3b–d) was demonstrated compared with the as-deposited CrN coating, which agreed well with calculation results of the grain size shown in Fig. 2b.

**Fig. 4:**
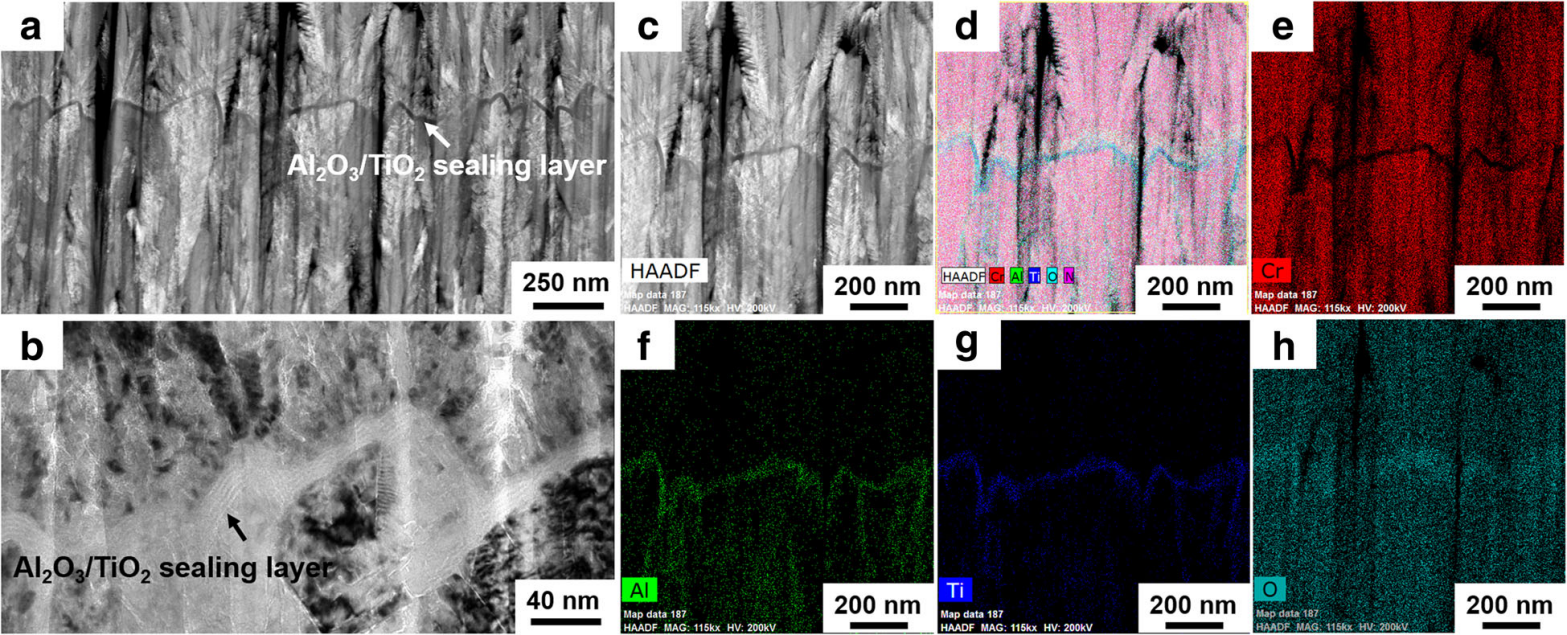
TEM/HRTEM/EDX analysis of the typical CrN-Al_2_O_3_/TiO_2_ hybrid coating: **a** low magnification bright field image. **b** HRTEM image of the nanolaminate-Al_2_O_3_/TiO_2_ sealing layer. **c-h** EDX mapping results of Cr, Al, Ti and O elements

To confirm the uniformity of the ALD sealing layer, the hybrid coatings of CrN-Al_2_O_3_/TiO_2_ were chosen for further investigation by TEM/HRTEM/EDX after FIB preparation, which was presented in Fig. 4. As it is evident from Fig. 4a, there was a strong contrast difference in the CrN matrix and the nanolaminate sealing layer. A clear line (~20 nm) with dark contrast was considered to belong to the nanolaminate-Al_2_O_3_/TiO_2_ sealing layer located in the CrN coatings (light contrast), where the uniform deposition of the ALD sealing layer on the rough surface of the first grown CrN could be confirmed evidently. In addition, a region with dense, small, and radiated new CrN sites was observed on the modified surface of the nanolaminate sealing layer, which was contributed to the nucleation of the new CrN grains at the initial stage of the second CrN deposition process. According to the HRTEM observations (Fig. 4b), the nanolaminate-Al_2_O_3_/TiO_2_ sealing layer, combing of two types of individual Al_2_O_3_ and TiO_2_ sub-layers stacking together, could be evidently distinguished by the gray and white contrast, indicating the success of the ALD deposition process and ideal design of this experiment. The EDX analysis of the sealing layer is shown in Fig. 4c–h. The Al and Ti existed exactly within the inner CrN matrix as a clear thin layer. Moreover, Al and Ti were also detected as penetrating into the CrN coating along the CrN grain boundary, indicating that the nanolaminate-Al_2_O_3_/TiO_2_ layer not only efficiently sealed the surface of the first-grown CrN coating but also extended into the pinhole defects of the CrN to completely cover the walls even with very narrow gaps, which is one of the most attractive advantages of ALD that can deposit ultrathin films on complicated high aspect ratio structures with excellent coverage [19].

**Fig. 5:**
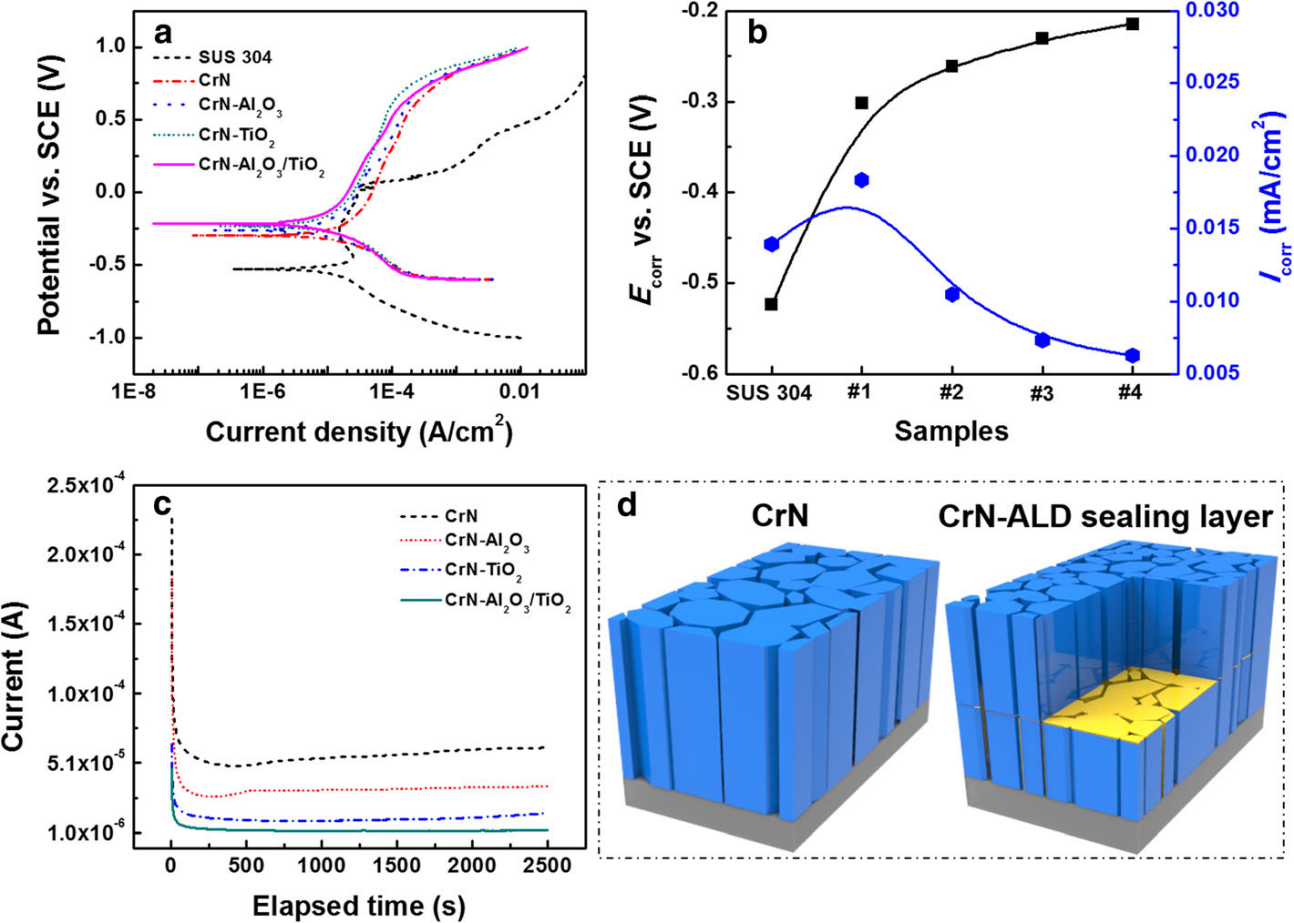
Potentiodynamic and potentiostatic curves of the SUS304 substrate, SUS304 substrate coated with CrN, CrN-Al_2_O_3_, CrN-TiO_2_ and CrN-Al_2_O_3_/TiO_2_ in 3.5 wt. % NaCl solution: **a** potentiodynamic curves. **b** calculation value of the corrosion potential and corrosion current density. **c** potentiostatic curves. **d** schematic illustration of corrosion behavior of the pure CrN coating and hybrid coating

Potentiodynamic and potentiostatic polarization tests were performed to investigate the corrosion behavior. Figure 5 shows the polarization curves, corrosion current density (*I*_corr_) values vs. the corrosion potentials (*E*_corr_), and the current density depending on the exposure time of the pure CrN and multilayered coatings. As seen in Fig. 5a–b, high potentials could be observed after applying the hybrid coatings compared with the bare SUS304 sample both without and with the CrN coating. The *I*_corr_ values were obtained from the Tafel plot by extending a straight line along the linear portion of the cathodic plot and extrapolating it to the *E*_corr_ axis due to the non-symmetry of the polarization curve between the anodic and cathodic branches [20–22]. An inverse relation existed between *I*_corr_ and *E*_corr_ through the quantitative analysis of the potentiodynamic curves; the *E*_corr_ continuously increased, while the *I*_corr_ increased slightly for the pure CrN coating and then it decreased with the ALD sealing layer in the CrN. The increased *I*_corr_ was considered due to the porous columnar CrN structure lead to some of the substrate exposing in the corrosion media. Therefore, a certain crevice corrosion rapidly would occur due to the localized attack during the corrosion investigation. And the final increase of the *I*_corr_ after ALD sealing layer applying indicated an improvement of corrosion resistance of the hybrid coatings resulting from the good passivation properties of the ALD sealing layers. The hybrid coatings with the nanolaminate-Al_2_O_3_/TiO_2_ sealing layers showed the best corrosion performance with the lowest *I*_corr_ of ~6.26 × 10^−6^ A/cm^2^ and highest *E*_corr_ of about −2.145 V, revealing that the nanolaminate Al_2_O_3_/TiO_2_ sealing layer proved to be more effective against corrosion in the corrosive media after applying the hybrid coatings. The current-time curves of CrN-Al_2_O_3_, CrN-TiO_2_, and CrN-Al_2_O_3_/TiO_2_ presented in Fig. 5c were acquired to verify the stability of the protection properties for a certain period at a given potential (*E* = 0.4 V vs. SCE) in the pitting region [12]. All of these curves showed a downward current density in the beginning. By comparison, the CrN exhibited a stable current density of approximately 5~6 × 10^−5^ A/cm^2^. However, after inserting the sealing layer, especially for the nanolaminate-Al_2_O_3_/TiO_2_, the lowest and most stable current density of 6~9 × 10^−6^ A/cm^2^ was achieved, reversing the good corrosion resistance of the coatings.

Based on the microstructure and corrosion performance analysis, the mechanism of the hybrid coating defense from the attack of the corrosive media was discussed. In previous works of an ALD interlayer inserted into HiPIMS-CrN coatings, the ALD interlayer was mostly deposited at the interface of the CrN due to the dense structure with a few tiny defects [12]. Here, the PVD-CrN presented a highly porous structure, leading to an ALD layer that not only deposited on the surface of the first CrN but also covered the side walls of its grain boundaries and the pinhole defects, as shown in the schematic in Fig. 5d. As a result, the consecutive sealing layer with low defects could act as a barrier layer for blocking the diffusion of corrosive substances by covering the pore walls of the CrN. Additionally, the ALD sealing layer with poor conductivity could improve the electro resistance of the protective coatings as an insulating layer. The further enhanced corrosion performance was attributed to the combination of Al_2_O_3_ and TiO_2_ as the sealing layers. Both Al_2_O_3_ and TiO_2_ are good protective coatings for engineering materials and offer good oxidation and corrosion resistance [4]. Compared to Al_2_O_3_, TiO_2_ displays much better water resistance from water corrosion than Al_2_O_3_. In this work, the combination of the TiO_2_ and Al_2_O_3_ nanolaminates with both lower resistance and anti-water corrosion as the sealing layer exhibited more effective performance than the single layer configuration, which was attributed to the synergistic effect of both Al_2_O_3_ and TiO_2_ [17, 23].

## Conclusions

In conclusion, CrN hybrid coatings were synthesized utilizing PVD and ALD techniques, and various sealing layers (Al_2_O_3_, TiO_2_, and Al_2_O_3_/TiO_2_) were inserted into the CrN matrix. By applying the ALD sealing layer, CrN coatings with denser microstructure were obtained, and the grain size of the coatings was decreased, while no change in the crystal structure was observed. The application of the ALD oxide sealing layer showed a positive effect on increasing the corrosion resistance of the CrN coatings. The ALD-TiO_2_ sealing layer indicated better corrosion resistance than the ALD-Al_2_O_3_ sealing layer. And the hybrid coatings with nanolaminate-Al_2_O_3_/TiO_2_ sealing layers showed the best corrosion performance with the highest corrosion potential and the lowest current density in both potentiodynamic and potentiostatic polarization tests. The enhanced corrosion performance of nanolaminate-Al_2_O_3_/TiO_2_ was considered to attribute to the synergistic effect of ALD-Al_2_O_3_ with high electrical resistivity and ALD-TiO_2_ with high stability in aqueous corrosive media. The results demonstrate that the nanolaminate ALD-Al_2_O_3_/TiO_2_ is a promising candidate for effective sealing layer against corrosion in harsh conditions.

## Abbreviations

AFM: Atomic force microscopy
ALD: Atomic layer deposition
CrN: Chromium nitride
CVD: Chemical vapor deposition
EDS: Energy dispersive spectrometer
FIB: Focused ion beam
HiPIMS: High power impulse magnetron sputtering
GPC: Growth rate per cycl﻿e
Pt: Platinum
PECVD: Plasma enhanced chemical vapor deposition
PVD: Physical vapor deposition
RMS: Root-mean-square
SCE: Saturated calomel electrode
SEM: Scanning electron microscopy
SUS: Stainless steels
Al(CH_3_)_3_: Trimethylaluminum
TEM: Transmission electron microscope
TTIP: Titanium isopropoxide
XRD: X-ray diffractometer
