# Erratum to: Enhanced Corrosion Resistance of PVD-CrN Coatings by ALD Sealing Layers

**DOI:** 10.1186/s11671-017-2079-8

**Published:** 2017-05-02

**Authors:** Zhixin Wan, Teng Fei Zhang, Ji Cheng Ding, Chang-Min Kim, So-Won Park, Yang Yang, Kwang-Ho Kim, Se-Hun Kwon

**Affiliations:** 1 The Institute of Materials Technology, Pusan National University, Busan, 46241 South Korea; 2 National Core Research Center for Hybrid Materials Solution, Pusan National University, Busan, 46241 South Korea; 3 School of Convergence Science, Pusan National University, Busan, 46241 South Korea; 4 School of Materials Science and Engineering, Pusan National University, Busan, 46241 South Korea; 5 State Key Laboratory of Materials-Oriented Chemical Engineering, College of Chemical Engineering, Nanjing Tech University, Nanjing, 210009 China

## Erratum

The name of the author [Zhixin Wan] was not completely displayed in the original publication [1]. The original article has been updated to rectify this error.
